# Hydrothermal synthesis of flower-like molybdenum disulfide microspheres and their application in electrochemical supercapacitors

**DOI:** 10.1039/c8ra04350g

**Published:** 2018-11-19

**Authors:** Fangping Wang, Guifang Li, Jinfeng Zheng, Jing Ma, Caixia Yang, Qizhao Wang

**Affiliations:** Key Laboratory of Eco-Environment-Related Polymer Materials, Ministry of Education of China, Key Laboratory of Gansu Polymer Materials, College of Chemistry and Chemical Engineering, Northwest Normal University Lanzhou 730070 China wangfp@nwnu.edu.cn

## Abstract

Three-dimensional flower-like molybdenum disulfide microspheres composed of nanosheets were prepared by a hydrothermal method using ammonium molybdate as the molybdenum source and thiourea as the sulfur source. Structural and morphological characterizations were performed by X-ray diffraction (XRD), scanning electron microscopy (SEM), transmission electron microscopy (TEM), energy-dispersive X-ray (EDX) spectroscopy and X-ray photoelectron spectroscopy (XPS). The electrochemical properties of MoS_2_ electrode were studied by performing cyclic voltammetry (CV), galvanostatic charge–discharge analysis and electrochemical impedance spectroscopy (EIS). When used as an electrode material for supercapacitor, the hybrid MoS_2_ showed a high specific capacity of 518.7 F g^−1^ at a current density of 1 A g^−1^ and 275 F g^−1^ at a high discharge current density of 10 A g^−1^. In addition, a symmetric supercapacitor composed of MoS_2_ as positive and negative electrodes was prepared, which exhibited a high energy density of 12.46 W h kg^−1^ at a power density of 70 W kg^−1^ and still maintains an impressive energy density of 6.42 W h kg^−1^ at a large power density of 7000 W kg^−1^. The outstanding performance of the MoS_2_ electrode material indicates its great potential for applications in high-performance energy storage systems.

## Abstract 2

Three-dimensional flower-like molybdenum disulfide microspheres composed of nanosheets were prepared by a hydrothermal method using ammonium molybdate as the molybdenum source and thiourea as the sulfur source.

## Graphical abstract

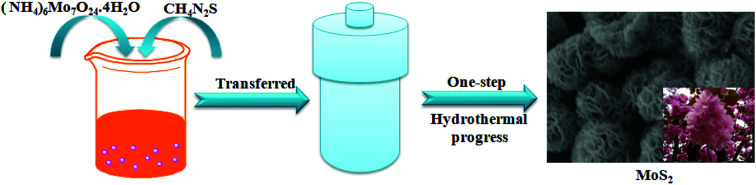

## 1. Introduction

Supercapacitors, also known as electrochemical capacitors, have gathered growing interest of researchers in the era of miniaturization of devices.^1,2^ These present fascinating properties of higher energy density, higher power density, longer life, lower toxicity than batteries, and so on, compared with those of traditional capacitors.^3–6^ According to the charge–discharge mechanisms, SCs can be divided into electrical double-layer capacitors (EDLCs) and pseudocapacitors.^7,8^ Pseudocapacitance arises from reversible faradaic reactions of redox active materials, such as transition metal oxides, hydroxides, and sulfides. Among those materials, ruthenium oxide (RuO_2_) has exhibited excellent pseudocapacitive performance, but the toxicity and high cost of RuO_2_ restrict its widespread commercial application.^9,10^ The low cost active material MnO_2_ can also achieve a high specific capacitance; however, MnO_2_-based pseudocapacitors suffer from poor electrical conductivity and cyclic stability.^11^ Application of nanometal sulfides in the energy storage devices, such as fuel cells, solar energy pools, lithium-ion batteries, and supercapacitors, have aroused widespread interest among researchers. At present, carbon materials (such as activated carbon), transition metal oxides (nickel oxide, *etc.*), and conductive polymers are often used as electrode materials for supercapacitors.^12,13^ However, the growing demand for energy storage devices has prompted researchers to develop new types of electrode materials. Therefore, the research of nanometer-scale metal sulfide as the material of supercapacitor electrode has become a new field. For example, cobalt sulfide (CoS, CoS_2_), nickel sulfide (NiS, NiS_2_, Ni_3_S_2_), molybdenum sulfide (MoS_2_), copper sulfide (CuS, Cu_2_S), and vanadium sulfide (VS, VS_2_) have been used as supercapacitors electrode materials.^14–16^ In particular, MoS_2_ has aroused interest among other transition metal sulfides due to its layered structure and inherent conductivity,^17^ and it is considered to be a suitable replacement for graphene and carbon nanotubes in energy storage applications. In addition, molybdenum-based materials (such as MoO_3_, MoO_2_, and MoS_2_) exhibit various valences and rich chemical properties, making them viable candidate materials for electrochemical applications.^18^

MoS_2_ is a transition metal sulfide with a layered structure, where a metal molybdenum layer is sandwiched between two sulfur layers; the layers are connected by weak van der Waals forces and the interlayer S–Mo–S atoms are strongly covalently linked.^19–21^ MoS_2_ possesses unique physicochemical properties due to its unique atomic and electronic structure. It is mainly used in the solid lubricants, catalysts, supercapacitors and lithium-ion batteries.^22–24^ Among these, the research on the application of MoS_2_ as a supercapacitor electrode material is the most extensive. For example, Soon *et al.*^25^ found that the MoS_2_ nano-film presented an electric double layer capacitance behavior. Ma *et al.*^26^ reported that nano-MoS_2_ intercalated in polypyrrole could improve its capacitance performance. Cao *et al.*^27^ fabricated micro-supercapacitors using coated MoS_2_ nanofilms, and showed that MoS_2_ has excellent electrochemical performance in aqueous electrolytes.

In particular, the structure of the electrode directly affects its electrochemical properties. Generally, the electrochemical electrode is 2-dimensional and suffers from inadequate contact with electrolyte and low surface-area-utilization efficiency. Numerous efforts have been made to design three-dimensional (3D) electrodes, such as MoS_2_/mesoporous carbon spheres. Recently, there have been some reports related to NiCo_2_S_4_ and graphene oxide composites applied in supercapacitors. Krishnamoorthy *et al.*^15^ reported 92.85 F g^−1^ specific capacitance of chemically prepared MoS_2_ nanostructure. Huang *et al.*^28^ reported polyaniline/MoS_2_ composites as supercapacitor electrodes with the specific capacitance of 575 F g^−1^.

**Fig. 1:**
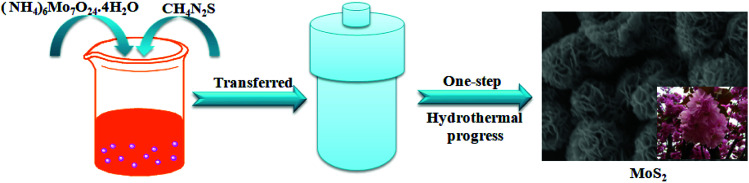
Schematic of the MoS_2_ synthesized by hydrothermal method.

In this paper, the morphologically regular flower-like molybdenum disulfide microspheres were successfully synthesized by a hydrothermal method (Fig. 1). The as-prepared MoS_2_ was directly used as a supercapacitor electrode and exhibited high specific capacitance (518.7 F g^−1^ at current density of 1 A g^−1^) and excellent cycling performance (88.2% retention after 2500 cycles). In addition, a high performance symmetric supercapacitor was successfully fabricated by using MoS_2_ as both positive electrode and negative electrode, which exhibited a high energy density of 12.46 W h kg^−1^ at power density of 70 W kg^−1^.

## 2. Experimental section

### 2.1. Materials

Ammonium molybdate ((NH_4_)_6_Mo_7_O_24_·4H_2_O) and thiourea (CH_4_N_2_S) were obtained from Tianjin Kaixin Chemical Industry Co. Ltd. All the chemical reagents were of analytical purity and used without any further purification.

### 2.2. Synthesis of MoS_2_

In a typical process, 0.8 g of ammonium molybdate and 5.12 g thiourea were dissolved into 80 mL deionized water and stirred until the solution was clear and transparent. The solution was transferred into 100 mL PTFE-lined stainless steel autoclave and heated at 200 °C for different time periods (8 h, 16 h, and 24 h). The obtained MoS_2_ was flushed with water and ethanol, in sequence, and then dried at 70 °C for 12 h. The MoS_2_ electrode materials were denoted as MoS_2_-8, MoS_2_-16, and MoS_2_-24, according to the hydrothermal treatment time.

### 2.3. Material characterization

The morphology and microstructure of the samples were characterized by field-emission scanning electron microscopy (FESEM JSM-6701F, Japan), transmission electron microscopy (TEM; JEOL, JEM-2010, Japan), and X-ray diffraction (XRD, D/Max-2400, Japan) with Cu Kα radiation (*λ* = 1.5418 Å) operating at 40 kV, 100 mA. X-ray photoelectron spectroscopy (XPS) spectra were recorded on a PHI 5702 spectrometer using a standard Al Kα X-ray source of 300 W and an analyser pass energy of 29.35 eV.

### 2.4. Electrode preparation and electrochemical characterization

The electrochemical properties of the MoS_2_ nanostructures were investigated in 1 M Na_2_SO_4_ solution using a three-electrode system in an electrochemical work station (CHI660E, Shanghai). Initially, 8 mg of MoS_2_-16 was dispersed in 400 μL of 0.5 wt% Nafion solution by ultrasonication to obtain a well dispersed suspension. Then, 6 μL of the suspension was drop-casted onto the pre-treated glassy carbon electrode (GCE) and left to dry at room temperature. Saturated calomel electrode, platinum wire, and a loadable glassy carbon electrode were respectively the reference, the counter, and the working electrodes.^29^ Cyclic voltammetry (CV) in the range −0.3 to 0.5 V was performed at different scan rates. Galvanostatic charge–discharge curves were recorded in the potential range of −0.3 to 0.5 V at different constant current density. The cycle life tests were performed by galvanostatic charge–discharge measurements with a constant current density of 4 A g^−1^ for 2500 cycles. Electrochemical impedance spectroscopy (EIS) was performed in the frequency range of 0.01 Hz to 100 kHz with 5 mV amplitude at current open circuit voltage.

A two-electrode symmetric supercapacitor cell was assembled to measure the device performances. MoS_2_ was used as the positive electrode and negative electrode. The negative electrode was prepared by the traditional slurry coating method. The mass loading of electroactive material in symmetric supercapacitor was 0.3 mg. The specific capacitances (*C*_m_) were calculated according to the following equations:^30–32^1
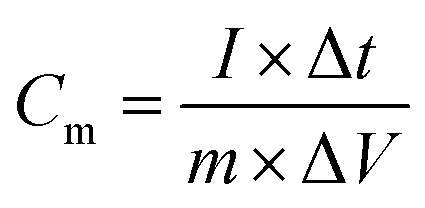
where *C*_m_ is the specific capacitance, *I* is the current of the charge–discharge, Δ*t* (s) is the discharge time, Δ*V* is the voltage window, and *m* is the mass of active materials.

In the symmetrical supercapacitors, the corresponding power density (*P*) and energy density (*E*) were calculated according to the following equations.^8^2
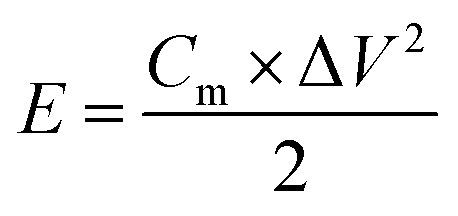
3
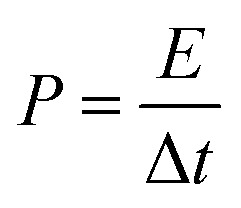


## 3. Results and discussion

### 3.1. Characterization of MoS_2_

**Fig. 2:**
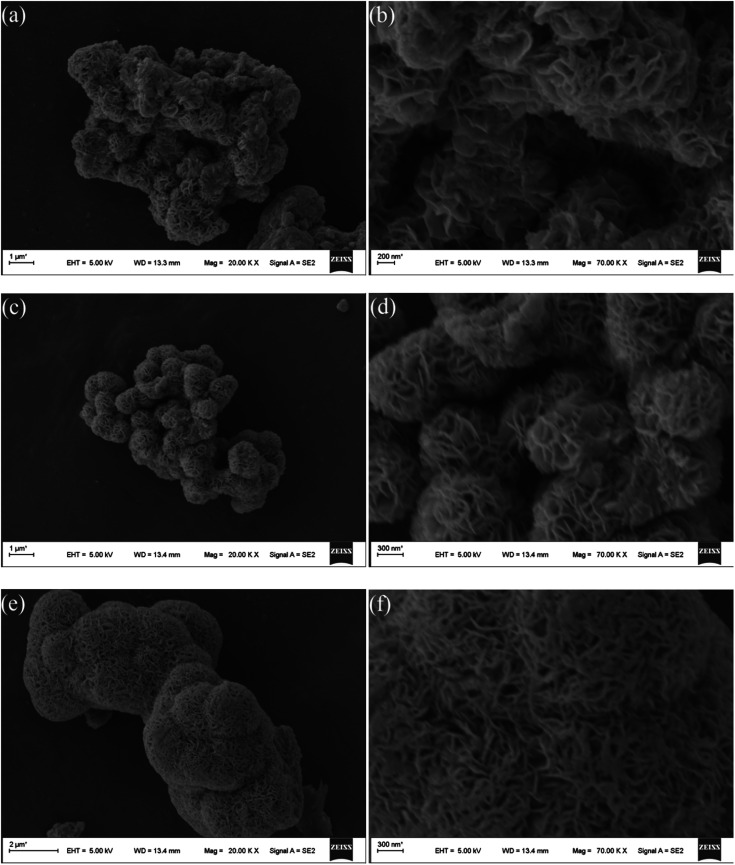
SEM images of (a and b) MoS_2_-8, (c and d) MoS_2_-16, and (e and f) MoS_2_-24.

Fig. 2 shows the SEM microstructures of the as-prepared MoS_2_-8, MoS_2_-16, and MoS_2_-24. It can be clearly observed from the Fig. 2 that the as-prepared molybdenum disulfide material has a nanoflower-like structure assembled from clear nanoflakes. Different hydrothermal treatment times had a great influence on the size of the molybdenum disulfide nanoflakes and the size of the three-dimensional pore structure. Fig. 2a and b show the SEM microstructures of the MoS_2_-8 at low and high magnifications, respectively. It can be seen that the nanosheets are partially adhered together and contained a small amount of block-like structures, resulting in inconspicuous pore structure. MoS_2_-16 (Fig. 2c and d) possesses an evenly distributed larger size of nanoflakes, and forms highly open and relatively deep porous nanostructures, making optimal use of the grain surface readily accessible to the liquid electrolyte and providing efficient channels for electron transport. Fig. 2e and f present the low and high magnification SEM image of the MoS_2_-24 sample. After a 24 h long hydrothermal process, the nanosheets of MoS_2_-24 arranged regularly but too tightly, and some collapsed, resulting in a decrease or disappearance of the pore size in the material, which could degrade the electrochemical performance of the electrode material.

**Fig. 3:**
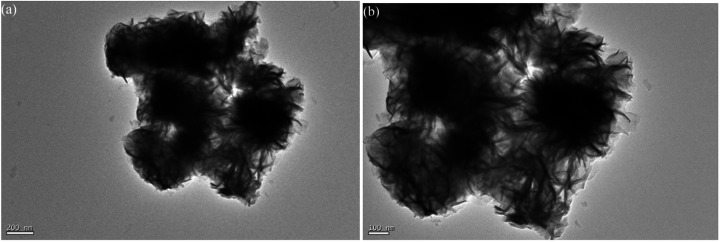
TEM images of MoS_2_-16.

Fig. 3 shows the TEM images for MoS_2_-16. As shown in Fig. 3a, the interconnected nanoflakes consist of nano-flowers. As seen in the magnified image (Fig. 3b), MoS_2_-16 nanoflakes are very thin, leading to open and porous three-dimensional structures, which are beneficial to electrolyte access and electron transport during electrochemical reactions. These results are in accordance with the SEM images.

**Fig. 4:**
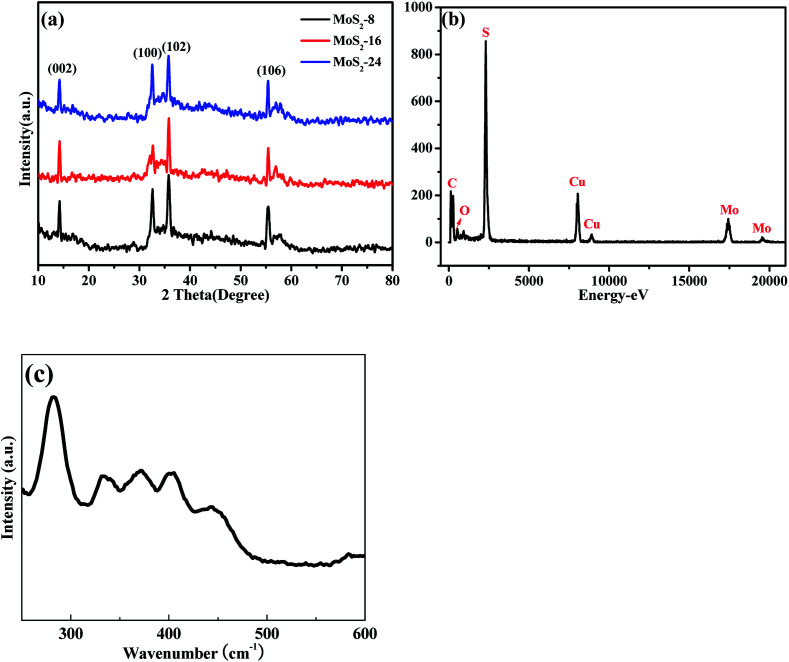
(a) XRD pattern of the MoS_2_-8, MoS_2_-16, and MoS_2_-24; (b) EDX spectra of MoS_2_-16; (c) Raman spectra of MoS_2_-16.

The XRD patterns of MoS_2_-8, MoS_2_-16, and MoS_2_-24 hybrids are shown in Fig. 4a. The four diffraction peaks at 14.2°, 32.5°, 35.8°, and 55.4° correspond to the (002), (100), (102) and (106) planes of cubic phase MoS_2_ (JCPDS No. 75-1539). Energy-dispersive X-ray (EDX) spectroscopy (Fig. 4b) demonstrates the existence of Mo and S elements. The Raman spectrum of the as-prepared MoS_2_ nanoflowers was recorded in this study, as shown in Fig. 4c. At low wave numbers the Raman spectrum of the MoS_2_ sample showed peaks at 145, 227, 283, 371 and 403 cm^−1^, related to the characteristic vibrations of pure metallic phase MoS_2_. The main peak associated with Mo–Mo metallic vibration is located at 145 cm^−1^. Two characteristic peaks are observed at 371 and 403 cm^−1^, which correspond to the E_2g_^1^ and A_g_^1^ modes of hexagonal MoS_2_, and are attributed to the out-of-plane Mo–S phonon mode and the in-plane Mo–S phonon mode, respectively.

**Fig. 5:**
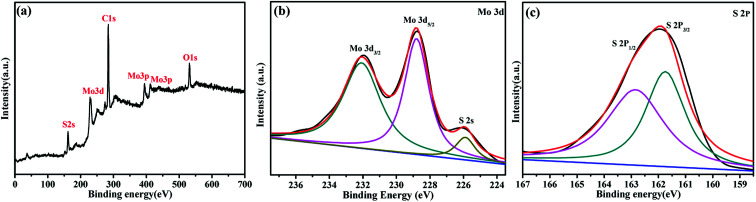
(a) XPS survey scanning of MoS_2_-16. XPS spectra of (b) Mo 3d and (c) S 2p.

The chemical and surface states of the Mo and S elements in the as-prepared MoS_2_-16 electrodes have been investigated *via* X-ray photoelectron spectroscopy. The XPS survey spectrum of the MoS_2_ electrodes is shown in Fig. 5a, which revealed the presence of Mo 3d, Mo 3p, S 2p, C 1s and O 1s states.^33^ The C and O signals originated from the CO_2_ and H_2_O impurities, as seen in many XPS analyses. The fine fitted spectrum of Mo 3d is shown in Fig. 5b, which revealed the presence of two major peaks at around 228.5 and 232 eV, corresponding to the Mo^4+^ 3d_5/2_ and Mo^4+^ 3d_3/2_ states, respectively. Small peaks belonging to S 2s in the vicinity of 226 eV are also observed.^34^ The fine fitted spectrum of S 2p (Fig. 5c) indicated the presence of two major peaks at around 161.5 and 162.9 eV, which corresponds to the S 2p_3/2_ and S 2p_1/2_ states, respectively.^35^ These studies confirm the formation of MoS_2_ by the hydrothermal method.

### 3.2. Electrochemical and energy storage performance

**Fig. 6:**
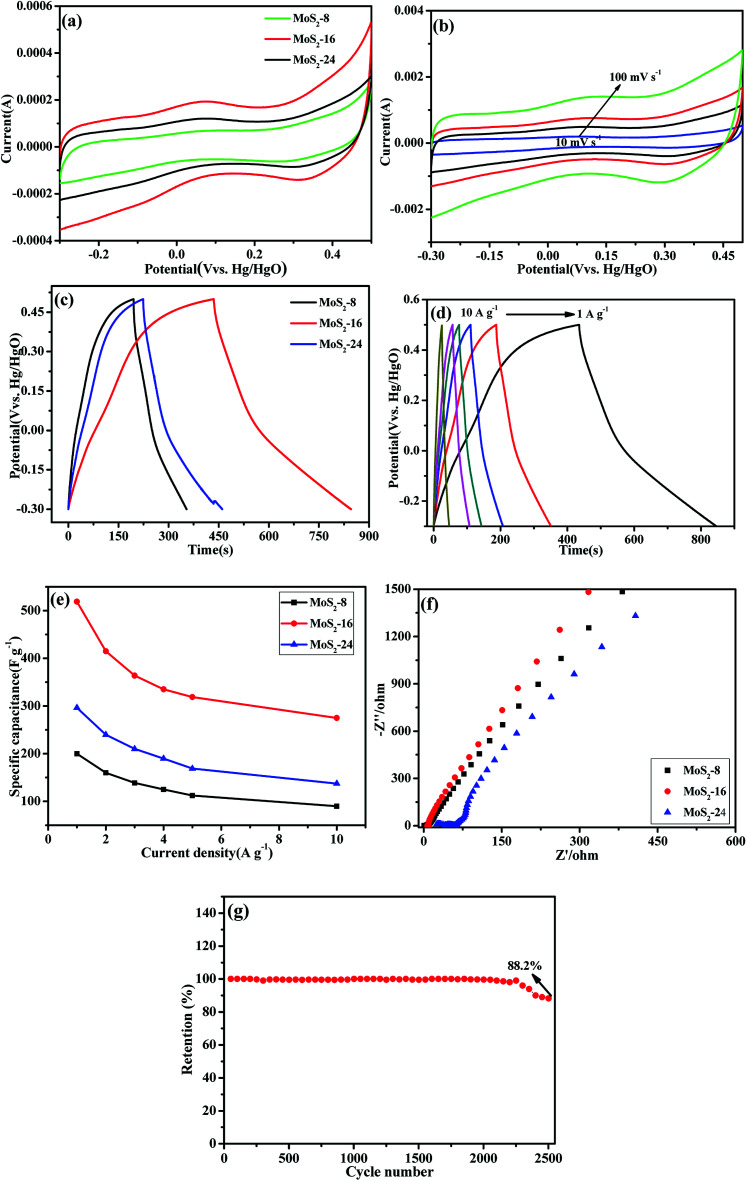
(a) Cyclic voltammograms of MoS_2_-8, MoS_2_-16, and MoS_2_-24 at 10 mV s^−1^. (b) CV curves of the MoS_2_-16 electrode at different scan rates. (c) Galvanostatic discharge curves of MoS_2_-8, MoS_2_-16 and MoS_2_-24 at a current density of 1 A g^−1^. (d) GCD curves of MoS_2_-16 at various current densities. (e) Specific capacitance as a function of the current density of the MoS_2_-8, MoS_2_-16, and MoS_2_-24. (f) Nyquist plots of MoS_2_-8, MoS_2_-16, and MoS_2_-24 electrodes in the frequency range from 100 kHz to 0.01 Hz. (g) Cycle performance for the MoS_2_-16 electrodes at a current of 4 A g^−1^.

Fig. 6a shows the cyclic voltammetry (CV) curves of MoS_2_-8, MoS_2_-16 and MoS_2_-24 at 10 mV s^−1^ in 1 M Na_2_SO_4_ solution, obtained over the potential range between −0.3 V and 0.5 V. In contrast, the CV curve area of the MoS_2_-16 electrode was larger than the electrode area of MoS_2_-8 and MoS_2_-24. Fig. 6b shows the cyclic voltammetry (CV) curves of MoS_2_-16 at different scan rates. On increasing the scanning speed from 10 mV s^−1^ to 100 mV s^−1^, the shape of the CV curve did not change significantly, indicating that MoS_2_-16 presented better rate performance and small polarization.^36,37^ Galvanostatic charging–discharging (GCD) technique was also applied to study the electrochemical capacitive properties of MoS_2_-8, MoS_2_-16 and MoS_2_-24 at a current density of 1 A g^−1^, as shown in Fig. 6c. The longer discharge time of MoS_2_-16 electrode again confirmed its enhanced capacitance. Fig. 6d shows the galvanostatic charge–discharge curve (GCD) of MoS_2_-16 at various current densities varying from 1 to 10 A g^−1^, with a potential window range from −0.3 V to 0.5 V. Based on eqn (2), for MoS_2_-16 electrode, at a discharge current of 1 A g^−1^, the specific capacitance reached 518.7 F g^−1^, while at a high discharge current of 10 A g^−1^, the specific capacitance was as high as 275 F g^−1^. Using these GCD curves, the specific capacitances of five electrodes at various current densities were calculated and depicted in Fig. 6e. The calculated specific capacitances of MoS_2_-16 electrode were calculated to be 518.7, 415, 363.7, 335, 318.7, and 275 F g^−1^ at discharge current densities of 1, 2, 3, 4, 5, and 10 A g^−1^, respectively, which are much higher than those for MoS_2_-8 and MoS_2_-24 at the same current densities. Table 1 compares the electrochemical performance of the MoS_2_ electrode material prepared in this study with that of the MoS_2_ electrode material reported in the literature. It can be seen that the electrochemical performance of the MoS_2_ electrode material prepared in this experiment is superior. The superior electrochemical behaviors of MoS_2_-16 nanoflower observed in this study should be partially attributed to its ultrathin and porous features, which can offer even richer electroactive sites, and more efficient and convenient electronic transport.

**Table 1:** Comparison of electrochemical properties of different MoS_2_ electrode materials

Samples	Electrolyte	Current density	Specific capacitance	References
MoS_2_ nanosheets	1 M Na_2_SO_4_	1 A g^−1^	129.2 F g^−1^	38
Sphere like MoS_2_	1 M Na_2_SO_4_	5 mV s^−1^	106 F g^−1^	39
Spherically clustered MoS_2_	1 M H_2_SO_4_	5 mV s^−1^	113 F g^−1^	40
MoS_2_ nanospheres	1 M KCl	1 A g^−1^	122 F g^−1^	41
Hollow MoS_2_ nanospheres	1 M KCl	0.59 A g^−1^	144 F g^−1^	42
MoS_2_ monolayers	6 M KOH	0.5 A g^−1^	366.9 F g^−1^	43
MoS_2_/CMG	1 M Na_2_SO_4_	0.5 A g^−1^	268 F g^−1^	44
MoS_2_/MWCNT	1 M Na_2_SO_4_	1 A g^−1^	452.7 F g^−1^	45
MoS_2_-16	1 M Na_2_SO_4_	1 A g^−1^	518.7 F g^−1^	This work

Electrochemical impedance spectroscopy (EIS) analysis is an important tool to examine the interface resistance of electrode materials for supercapacitors. For an ideal supercapacitor, the Nyquist plot comprises a vertical line, which can be simulated by an equivalent circuit. The semicircle at high frequency region is indicative of interfacial charge transfer resistance. In the equivalent circuit, the series resistance (*R*) depends on electrolyte resistance and electrode electronic resistance. Nyquist plots based on the radius of the high frequency arc on the real axis are shown in Fig. 6f. Clearly, the semicircle over the high frequency range of the MoS_2_-16 electrode is smaller than that of others, indicating the smaller charge-transfer resistance. Furthermore, the slope of the line for MoS_2_-16 was larger than that of MoS_2_-8 and MoS_2_-24, implying a better capacitive behavior and a lower diffusion resistance of ions in the MoS_2_-16 electrode material. The differences in the electrochemical properties of MoS_2_ material are mainly due to disparity in the material electrolyte interface properties and electrolyte ion diffusion rates during the charge–discharge processes, which are in good accordance with its abovementioned electrochemical performance.

The cyclic stability of the electrode material is very important for practical supercapacitor applications. The cycling performance of MoS_2_-16 electrode was tested by 2500 cycles of continuous galvanostatic charge/discharge at the current density of 4 A g^−1^ (Fig. 6g). Although the specific capacitance gradually decreases with the increase of cycle number, there is still 88.2% retention of the initial capacitance.

### 3.3. Electrochemical performances of the MoS_2_-16//MoS_2_-16 symmetric supercapacitor

**Fig. 7:**
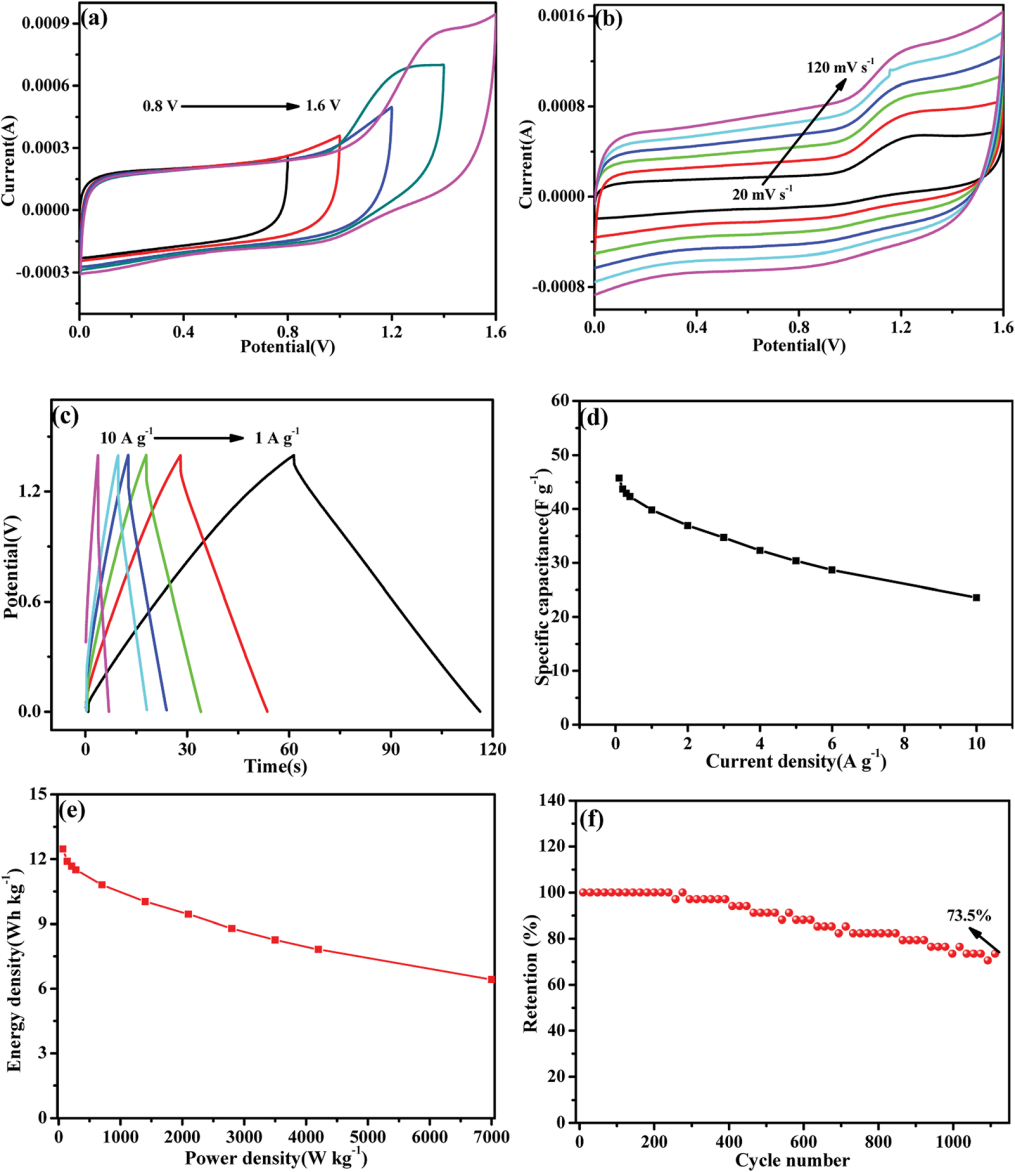
(a) CV curves of MoS_2_-16//MoS_2_-16 measured at different potential windows at a scan rate of 30 mV s^−1^. (b) CV curves of the SC measured at different scan rates ranging from 20 to 120 mV s^−1^ in potential window of 0 to 1.6 V. (c) Galvanostatic charge/discharge curves of the SC measure at different current densities from 1 to 10 A g^−1^. (d) Specific capacitance as a function of the current density of the symmetric supercapacitor. (e) Ragone plots of the SC. (f) Cycling stability of the SC at 1.6 A g^−1^.

To further evaluate the practical application potential of MoS_2_-16 electrode, an aqueous SC was first assembled using the MoS_2_-16 electrode as both positive electrode and negative electrode. Fig. 7a shows a series of CV curves collected at 30 mV s^−1^ with an operating SC voltage ranging from 0.8 to 1.6 V to obtain the best operating potential of MoS_2_-16//MoS_2_-16. Fig. 7b shows typical CV curves for the SC device corresponding to different sweep rates. With the increment of sweep rate from 20 to 120 mV s^−1^, all the curves presented similar shapes, revealing the splendid high-rate charge–discharge performance of the device.^46–48^Fig. 7c shows the typical GCD curves of the cells at various current densities with a potential window of 0–1.4 V. During the charge and discharge processes, the charge curve of MoS_2_-16//MoS_2_-16 (SSC) and its corresponding discharge curve are observed to be symmetrical, confirming that it has excellent electrochemical reversibility.^49^ The calculated specific capacitance values based on the discharge curves are plotted in Fig. 7d, which are 45.7, 43.7, 42.8, 42.3, 39.8, 36.9 and 23.57 F g^−1^ at 0.1, 0.2, 0.3, 0.4, 1, 2, and 10 A g^−1^, respectively. Energy density and power density are important parameters to evaluate the performance of symmetric supercapacitors. Fig. 7e presents the Ragone plot of the as-fabricated MoS_2_-16//MoS_2_-16 symmetric device, which describes the relationship between the energy and power densities. The supercapacitor device delivered a high energy density of 12.46 W h kg^−1^ at the power density of 70 W kg^−1^ and still maintained 6.42 W h kg^−1^ at 7 kW kg^−1^. Previously reported literature also reported energy storage tests on these similar electrode materials. The as-fabricated self-charging supercapacitor power cell (SCSPC) delivered a specific capacitance of 18.93 mF cm^−2^ with a specific energy of 37.90 mJ cm^−2^ at a specific power density of 268.91 μW cm^−2^, which were obtained at a constant discharge current of 0.5 mA.^50^ For the s-MoS_2_/CNS-based symmetric pseudocapacitor, the equivalent values were 108 F g^−1^, 7.4 W h kg^−1^ and 3700 W kg^−1^.^51^ The MoS_2_-based wire-type solid state supercapacitors (WSCs) device delivered a specific capacitance of 119 μF cm^−1^, and energy density of 8.1 nW h cm^−1^.^52^ Furthermore, the cycling stability of the as-fabricated SC was performed by repeating the GCD test at a current density of 1.6 A g^−1^. The specific capacitance retention of MoS_2_-16//MoS_2_-16 was about 73.5% after 1100 cycles, revealing that this symmetric supercapacitor has eminent cycling stability.

## 4. Conclusions

In summary, we have designed and successfully fabricated flower-shaped MoS_2_ microspheres assembled from many nanoflakes. The capacitive properties of flower-shaped molybdenum disulfide microspheres as the material of the supercapacitor electrode were studied. The obtained MoS_2_-16 nanoflower delivered a high specific capacitance of 518.7 F g^−1^ at a current density of 1 A g^−1^, with capacitance retention of 88.2% after 2500 cycles in alkaline system in a three-electrode cell. To further confirm its practicability, an symmetric supercapacitor was assembled using the MoS_2_-16 nanoflower as both positive electrode and negative electrode. This supercapacitor delivered a maximum energy density of 12.46 W h kg^−1^ at a power density of 70 W kg^−1^. Even at the highest power density of 7000 W kg^−1^, the MoS_2_-16//MoS_2_-16 device still maintained an energy density of 6.42 W h kg^−1^. Such outstanding capacitive behaviors imply the MoS_2_-16 nanoflower as a promising material for energy storage devices.

## Conflicts of interest

There are no conflicts to declare.

## Supplementary Material

## References

[cit1] Javed M. S., Dai S., Wang M., Guo D., Chen L., Wang X., Hu C., Xi Y. (2015). High performance solid state flexible supercapacitor based on molybdenum sulfide hierarchical nanospheres. J. Power Sources.

[cit2] Xie X. C., Huang K. J., Wu X. (2018). Metal-organic framework derived hollow materials for electrochemical energy storage. J. Mater. Chem. A.

[cit3] Zhai Z. B., Huang K. J., Wu X. (2018). Superior mixed Co-Cd selenide nanorods for high performance alkaline battery-supercapacitor hybrid energy storage. Nano Energy.

[cit4] Guana C., Liu J., Cheng C., Li H., Li X., Zhou W., Zhang H., Fan H. (2011). Hybrid structure of cobalt monoxide nanowire @ nickel hydroxidenitrate nanoflake aligned on nickel foam for high-rate supercapacitor. Energy Environ. Sci..

[cit5] Su Z., Yang C., Xie B., Lin Z., Zhang Z., Liu J., Li B., Kang F., Wong C. (2014). Scalable fabrication of MnO_2_ nanostructure deposited on free-standing Ni nanocone arrays for ultrathin, flexible, high-performance micro-supercapacitor. Energy Environ. Sci..

[cit6] Wu H., Lou Z., Yang H., Shen G. (2015). A flexible spiral-type supercapacitor based on ZnCo_2_O_4_ nanorod electrodes. Nanoscale.

[cit7] Zou R., Yuen M. F., Zhang Z., Hu J., Zhang W. (2015). Three-dimensional networked NiCo_2_O_4_/MnO_2_ branched nanowire heterostructure arrays on nickel foam with enhanced supercapacitor performance. J. Mater. Chem. A.

[cit8] Li R., Wang S., Huang Z., Lu F., He T. (2016). NiCo_2_S_4_@Co(OH)_2_ core-shell nanotube arrays in situ grown on Ni foam for high performances asymmetric supercapacitors. J. Power Sources.

[cit9] Hu C. C., Chang K. H., Lin M. C., Wu Y. T. (2006). Design and tailoring of the nanotubular arrayed architecture of hydrous RuO_2_ for next generation supercapacitors. Nano Lett..

[cit10] Lin J. Y., Chou S. W. (2013). Cathodic deposition of interlaced nanosheet-like cobalt sulfide films for high-performance supercapacitors. RSC Adv..

[cit11] Wang Z., Zhu J., Sun P., Zhang P., Zeng Z., Liang S., Zhu X. (2014). Nanostructured Mn–Cu binary oxides for supercapacitor. J. Alloys Compd..

[cit12] Karthikeyan K., Antony A., Sun M. Y., Jae K. S. (2014). Plasma assisted synthesis of graphene nanosheets and their supercapacitor applications. Sci. Adv. Mater..

[cit13] Wu H. B., Pang H., Lou X. W. (2013). Facile synthesis of mesoporous Ni_0.3_Co_2.7_O_4_ hierarchical structures for high-performance supercapacitors. Energy Environ. Sci..

[cit14] Krishnamoorthy K., Veerasubramanik G. K., Radhakrishnan S., Kim S. J. (2014). One pot hydrothermal growth of hierarchical nanostructured Ni_3_S_2_ on Ni foam for supercapacitor application. Chem. Eng. J..

[cit15] Krishnamoorthy K., Veerasubramani G. K., Radhakrishnan S., Kim S. J. (2014). Supercapacitive properties of hydrothermally synthesized sphere like MoS_2_ nanostructures. Mater. Res. Bull..

[cit16] Zhang W. J., Huang K. J. (2017). A review of recent progress in molybdenum disulfide-based supercapacitors and batteries. Inorg. Chem. Front..

[cit17] Krishnamoorthy K., Veerasubramani G. K., Pazhamalai P., Kim S. J. (2016). Designing two dimensional nanoarchitectured MoS_2_ sheets grown
on Mo foil as a binder free electrode for supercapacitors. Electrochim. Acta.

[cit18] Hu X., Zhang W., Liu X., Meia Y., Huang Y. (2015). Nanostructured Mo-based electrode materials for electrochemical energy storage. Chem. Soc. Rev..

[cit19] Wang L., Xu Z., Wang W., Bai X. (2014). Atomic mechanism of dynamic electrochemical lithiation processes of MoS_2_ nanosheets. J. Am. Chem. Soc..

[cit20] Zhao X., Sui J., Li F., Fang H., Wang H., Li J., Cai W., Cao G. (2016). Lamellar MoSe_2_ nanosheets embedded with MoO_2_ nanoparticles: novel hybrid nanostructures promoted excellent performances for lithium ion batteries. Nanoscale.

[cit21] Shi Y., Zhou W., Lu A. Y., Fang W., Lee Y. H., Hsu A. L., Kim S. M., Kim K. K., Yang H. Y., Li L. J., Idrobo J. C., van der J. K. (2012). Waals epitaxy of MoS_2_ layers using graphene as growth templates. Nano Lett..

[cit22] Lee Y. H., Zhang X. Q., Zhang W., Chang M. T., Lin C. T., Chang K. D., Yu Y. C., Wang J. W., Chang C. S., Li L. J., Lin T. W. (2012). Synthesis of large-area MoS_2_ atomic layers with chemical vapor deposition. Adv. Mater..

[cit23] Acerce M., Voiry D., Chhowalla M. (2015). Metallic 1T phase MoS_2_ nanosheets as supercapacitor electrode materials. Nat. Nanotechnol..

[cit24] Bindumadhavan K., Srivastava S. K., Mahanty S. (2013). MoS_2_-MWCNT hybrids as a superior anode in lithium-ion batteries. Chem. Commun..

[cit25] Soon J. M., Loh K. P. (2007). Electrochemical double-layer capacitance of MoS_2_ nanowall films. Electrochem. Solid-State Lett..

[cit26] Ma G., Peng H., Mu J., Huang H., Zhou X., Lei Z. (2013). In situ intercalative polymerization of pyrrole in graphene analogue of MoS_2_ as advanced electrode material in supercapacitor. J. Power Sources.

[cit27] Cao L., Yang S., Gao W., Liu Z., Gong Y., Ma L., Shi G., Lei S., Zhang Y., Zhang S., Vajtai R., Ajayan P. M. (2013). Direct laser-patterned micro-supercapacitors from paintable MoS_2_ films. Small.

[cit28] Huang K. J., Wang L., Liu Y. J., Wang H. B., Liu Y. M., Wang L. L. (2013). Synthesis of polyaniline/2-dimensional graphene analog MoS_2_ composites for high-performance supercapacitor. Electrochim. Acta.

[cit29] Sarno M., Galvagno S., Piscitelli R., Portofino S., Ciambelli P. (2016). Supercapacitor Electrodes Made of Exhausted Activated Carbon-Derived SiC Nanoparticles Coated by Graphene. Ind. Eng. Chem. Res..

[cit30] Wang X., Xiao Y., Su D., Zhou L., Wu S., Han L., Fang S., Cao S. (2016). High-quality Porous Cobalt Monoxide Nanowires@Ultrathin Manganese dioxide Sheets Core-Shell Nanowire Arrays on Ni Foam for High-Performance Supercapacitor. Electrochim. Acta.

[cit31] Yang P., Main W. (2014). Flexible solid-state electrochemical supercapacitors. Nano Energy.

[cit32] Xing L. L., Huang K. J., Cao S. X., Pang H. (2018). Chestnut shell-like Li_4_Ti_5_O_12_ hollow spheres for
high-performance aqueous asymmetric supercapacitors. Chem. Eng. J..

[cit33] Pu Z., Liu Q., Asiri A. M., Luo Y., Sun X., He Y. (2015). 3D macroporous MoS_2_ thin film: in situ hydrothermal preparation and application as a highly active hydrogen evolution electrocatalyst at all pH values. Electrochim. Acta.

[cit34] Shi J., Yang Y., Zhang Y., Ma D., Wei W., Ji Q., Zhang Y., Song X., Gao T., Li C., Bao X., Liu Z., Fu Q., Zhang Y. (2015). Monolayer MoS_2_ growth on Au foils and on-site domain boundary imaging. Adv. Funct. Mater..

[cit35] Lu Z., Zhang H., Zhu W., Yu X., Kuang Y., Chang Z., Lei X., Sun X. (2013). In situ fabrication of porous MoS_2_ thin-films as high-performance catalysts for electrochemical hydrogen evolution. Chem. Commun..

[cit36] Yuan C., Li J., Hou L., Zhang X., Shen L., Lou X. W. (2012). Ultrathin mesoporous NiCo_2_O_4_ nanosheets supported on Ni foam as advanced electrodes for supercapacitors. Adv. Funct. Mater..

[cit37] Wei J. J., Liu H., Niki K., Margoliash E., Waldeck D. H. (2004). Probing electron tunneling pathways: electrochemical study of rat heart cytochrome c and its mutant on pyridine-terminated SAMs. J. Phys. Chem. B.

[cit38] Huang K. J., Zhang J. Z., W Shi G., Liu Y. M. (2014). Hydrothermal synthesis of molybdenum disulfide nanosheets as supercapacitors electrode material. Electrochim. Acta.

[cit39] Karthikeyan K., Veerasubramani G. K., Radhakrishnan S., Kim S. J. (2014). Supercapacitive properties of hydrothermally synthesized sphere like MoS_2_ nanostructures. Mater. Res. Bull..

[cit40] Ilanchezhiyan P., Mohan Kumar G., Kang T. W. (2015). Electrochemical studies of spherically clustered MoS_2_ nanostructures for electrode applications. J. Alloys Compd..

[cit41] Zhou X., Xu B., Lin Z., Shu D., Ma L. (2014). Hydrothermal synthesis of flower-like MoS_2_ nanospheres for electrochemical supercapacitors. J. Nanosci. Nanotechnol..

[cit42] Wang L., Ma Y., Yang M., Qi Y. (2015). Hierarchical hollow MoS_2_ nanospheres with enhanced electrochemical properties used as an electrode in supercapacitor. Electrochim. Acta.

[cit43] Jiang L., Zhang S., Kulinich S. A., Song X., Zhu J., Wang X., Zeng H. (2015). Optimizing hybridization of 1T and 2H phases in MoS_2_ monolayers to improve capacitances of supercapacitors. Mater. Res. Lett..

[cit44] Yang M. H., Jeong J. M., Huh Y. S., Choi B. G. (2015). High-performance supercapacitor based on three-dimensional MoS_2_/graphene aerogel composites. Compos. Sci. Technol..

[cit45] Huang K. J., Wang L., Zhang J. Z., Wang L. L., Mo Y. P. (2014). One-step preparation of layered molybdenum disulfide/multi-walled carbon nanotube composites for enhanced performance supercapacitor. Energy.

[cit46] Liu S., Hui K. S., Hui K. N. (2016). Flower-like copper cobaltite nanosheets on graphite paper as high-performance supercapacitor electrodes and enzymeless glucose sensors. ACS Appl. Mater. Interfaces.

[cit47] Pan X., Ren G., Hoque M. N. F., Bayne S., Zhu K., Fan Z. (2014). Fast supercapacitors based on graphene-bridged V_2_O_3_/VO_x_ core-shell nanostructure electrodes with a power density of 1 MW kg^-1^. Adv. Mater. Interfaces.

[cit48] Wang F., Li G., Zheng J., Ma J., Yang C., Wang Q. (2018). Microwave synthesis of three-dimensional nickel cobalt sulfide nanosheets grown on nickel foam for high-performance asymmetric supercapacitors. J. Colloid Interface Sci..

[cit49] Cheng S., Shi T., Huang Y., Tao X., Li J., Cheng C., Liao G., Tang Z. (2017). Rational design of nickel cobalt sulfide/oxide core-shell nanocolumn arrays for high-performance flexible all-solid-state asymmetric supercapacitors. Ceram. Int..

[cit50] Parthiban P., Krishnamoorthy K., Mariappan V. K., Sahoo S., Manoharan S., Kim S. (2018). A high efficacy self-charging MoSe_2_ solid-state supercapacitor using electrospun nanofibrous piezoelectric separator with ionogel electrolyte. Adv. Mater. Interfaces.

[cit51] Khawula T. N. Y., Raju K., Franklyn P. J., Sigalas I., Ozoemena K. I. (2016). Symmetric pseudocapacitors based on molybdenum disulfide (MoS_2_)-modified carbon nanospheres: correlating physicochemistry and synergistic interaction on energy storage. J. Mater. Chem. A.

[cit52] Karthikeyan K., Pazhamalai P., Veerasubramani G. K., Kim S. J. (2016). Mechanically delaminated few layered MoS_2_ nanosheets based high performance wire type solid-state symmetric supercapacitors. J. Power Sources.

